# Typical and atypical metabolic characteristics of three iridaceae isoflavone components: *in vitro* and *in silico* studies

**DOI:** 10.3389/fphar.2025.1522857

**Published:** 2025-02-11

**Authors:** Jifeng Gu, Huishan Zhang, Mei Wang, Yuyang Zhou, Zhipeng Deng, Rong Shi

**Affiliations:** ^1^ Department of Pharmacy, Eye and ENT Hospital, Fudan University, Shanghai, China; ^2^ Shanghai Key Laboratory of Bioactive Small Molecules, School of Basic Medical Sciences, Fudan University, Shanghai, China; ^3^ Science and Technology Experimental Center, Shanghai University of Traditional Chinese Medicine, Shanghai, China; ^4^ Department of Pharmacology, Addiction Science, Toxicology, College of Medicine, University of Tennessee Health Science Center, Memphis, TN, United States; ^5^ School of Pharmacy, Shandong University of Traditional Chinese Medicine, Jinan, China

**Keywords:** tectorigenin, irigenin, irisflorentin, atypical kinetics, metabolism, human liver microsomes

## Abstract

**Background:**

*Belamcanda chinensis* (L.) DC (Chinese name Shegan) has been widely used because of its pharmacological activity and remarkable therapeutic effects in sore throat. Tectorigenin, irigenin, and irisflorentin have been recognized as important quality indicators in Shegan. However, the metabolic characteristics of isoflavone aglycones remain unclear.

**Methods:**

In this study, human liver microsomes (HLMs) and Cytochrome P450 (CYP) recombinant enzymes were used to study the metabolic stability, identify the metabolic pathways and enzyme kinetics of these three components, and elucidate their possible binding sites through molecular docking.

**Results:**

When tectorigenin, irigenin, and irisflorentin were co-incubated with HLMs and CYP recombinant enzymes, hydroxylation metabolite for tectorigenin, demethylated metabolite for irigenin, and 6,7-dihydroxy-5,3′,4′,5′-tetramethoxy isoflavone originating from irisflorentin were identified. CYP2E1 and CYP3A4 have high metabolic rates for tectorigenin, whereas CYP2C19 and CYP1A2 are the most important metabolic enzymes for irigenin and irisflorentin, respectively. The kinetics showed that the metabolism of tectorigenin and irigenin conformed to the Michaelis-Menten model, while the Eadie-Hofstee plot of irisflorentin yielded a convex curve with a unique “hooked” characteristic, and it conformed to the sigmoidal kinetics characteristic. Furthermore, molecular simulations showed that tectorigenin and irigenin bind to the orthosteric site of CYP isoforms via hydrogen bonds and π-π stacking, whereas irisflorentin is principally bound to CYP1A2 via π-π stacking and hydrophobic interactions.

**Conclusion:**

Collectively, these *Iridaceae isoflavone* aglycones can be metabolized by CYP enzymes with typical or atypical kinetic characteristics. These results lay a foundation for a better understanding of the *in vivo* processes of these components.

## 1 Introduction

*Belamcanda chinensis* (L.) DC (Chinese name Shegan) is an herb belonging to the genus Belamcanda of the Iridaceae family (Xiong et al., 2024). It is effective in clearing heat, detoxifying and eliminating phlegm, and soothing the throat. Among the prescriptions for treating throat disorders in the book “Li Dai Ming Yi Liang Yao Zhu Shi”, one-fourth of the prescriptions contain Shegan, which is often the principal Chinese medicine. Pharmacological studies have found Shegan exerts various pharmacological effects, including significant anti-inflammatory, analgesic, and antibacterial effects.

The medicinal efficacy of Shegan can be attributed to its rich isoflavone content. Multiple studies have confirmed that isoflavones, including tectorigenin (Rong et al., 2023), irigenin (Guo et al., 2020; Xu et al., 2022), and irisflorentin (Patel, 2023), exhibit anti-inflammatory, anti-oxidation, and anticancer activities. These components may serve as important bioactive markers in Shegan (Wang et al., 2024). Therefore, it is necessary to gain an in-depth understanding of the processes of these components *in vivo*, particularly their metabolic characteristics.

The Cytochrome 450 (CYP) superfamily of enzymes serves as the principal phase I enzyme in the metabolism of diverse exogenous, endogenous, and herbal compounds (Zhang X. et al., 2021). Research on the phase I metabolism of tectorigenin, irigenin, and irisflorentin is limited. Researchers (Shi et al., 2015; Wang et al., 2013) have found that tectorigenin is widely metabolized *in vivo*; however, the metabolizing enzymes that catalyze its metabolism are still unclear, and the enzymatic kinetic characteristics have not been reported. As an index component of Shegan in the Pharmacopoeia of the People’s Republic of China, irisflorentin could be metabolized by rat liver microsomes (Zhang Y. et al., 2021); however, its enzymatic kinetic characteristic is currently unknown.

Therefore, in this study, human liver microsomes (HLMs) was used to observe the phase I metabolites of three isoflavone compounds, investigated the subtypes of enzymes involved in metabolism using chemical inhibitors and recombinant enzymes, conducted enzymatic kinetics studies, and explained their possible binding sites through molecular docking. These results lay a understanding of the *in vivo* processes of these three compounds and provide the foundation for further study.

## 2 Materials and methods

### 2.1 Materials

Nicotinamide adenine dinucleotide phosphate tetrasodium salt (NADPH), α-naphthoflavone (α-Naph), tryptamine (Tryp), sulfaphenazole (Spz), ticlopidine hydrochloride (Ticlo), diethyldithiocarbamate (DDC), quinidine (Qd), and ketoconazole (KCZ) were obtained from Sigma-Aldrich (St. Louis, MO, United States). HLMs (protein concentration: 15 mg/mL, mixed sex) were obtained from Rild-Biotech Co. Ltd (Shanghai, China). Recombinant enzymes CYP1A2, CYP2A6, CYP2C8, CYP2C9, CYP2C19, CYP2D6, CYP2E1, and CYP3A4 were acquired from CYPEX (Dundee, United Kingdom). Tectorigenin, irigenin, and irisflorentin (>98% purity) were acquired from Yuanye Biotechnology Co. Ltd (Shanghai, China). All other analytically pure reagents were sourced from Sinopharm Group Co., Ltd (Shanghai, China).

### 2.2 Metabolic stability study in HLMs

To analyze the metabolic stability, a phase I metabolic system was established. Tectorigenin, irigenin, or irisflorentin (100 μM) was pre-incubated at 37°C with Tris-HCl buffer solution (50 mM), MgCl_2_ (10 mM), and HLMs (0.5 mg protein/mL). After 5 min, NADPH (1 mM) was added to start the metabolic reactions. After incubation for 30 min, an equal volume of methanol was added to terminate the reaction. After centrifugation, the supernatant was used for metabolite analysis. By comparing the high-performance liquid chromatography (HPLC) chromatograms of the samples incubated for 0 and 30 min to determine the generation of metabolites, mass spectrometry information for the metabolites was obtained using an UPLC-linear ion Trap-Orbitrap Mass Spectrometry method.

### 2.3 Inhibition by chemical inhibitors

To investigate the role of specific CYP subtypes in metabolism, tectorigenin, irigenin, or irisflorentin (100 μM) was co-incubated with HLMs (0.5 mg protein/mL), NADPH (1 mM), and MgCl_2_ (10 mM) in the presence of known CYP inhibitors. The inhibitors used were α-Naph (20 μM) for CYP1A2 (Zhang Y. J. et al., 2021), Tryp (2 μM) for CYP2A6 (Rodríguez-Morató et al., 2017), Spz (10 μM) for CYP2C9 (Berger et al., 2018), Ticlo (2 μM) for CYP2B6 and 2C19 (Yang et al., 2019), Qd (8 μM) for CYP2D6 (Martin et al., 2022), DDC (100 μM) for CYP2E1 (Zhang X. et al., 2021), and KCZ (1 μM) for CYP3A4 (Berger et al., 2018). Finally, the samples obtained by centrifugation after adding ice-cold methanol to the incubated samples were analyzed using HPLC. Samples without inhibitors were used as controls to calculate the inhibition rates. Each inhibitor group and the control group were compared. All experiments were carried out in triplicate.

### 2.4 Reaction phenotyping

To assess the role of CYP enzymes to isoflavone metabolism, tectorigenin, irigenin, or irisflorentin (100 μM) was co-incubated with 25 pmol of recombinant CYP enzymes (CYP1A2, CYP2A6, CYP2C8, CYP2C9, CYP2C19, CYP2D6, CYP2E1, and CYP3A4) and MgCl_2_ (10 mM) in Tris-HCl buffer. The samples were pre-incubated at 37°C for 5 min, and the reaction was initiated by the addition of NADPH. After 30 min of incubation, the supernatant obtained by centrifugation after adding methanol to the incubated sample was analyzed by HPLC. All experiments were carried out in triplicate.

### 2.5 Enzyme kinetics experiment

A series of concentrations (1–300 μM) of tectorigenin, irigenin, or irisflorentin were added to HLMs or recombinant enzymes. Nonlinear regression was performed on the substrate concentration and generation rate of metabolites to investigate the enzymatic kinetic characteristics of these components in the metabolic system. All experiments were carried out in triplicate.

### 2.6 Quantitative analysis of the metabolites

A Waters AQUITY UPLC system (Waters, Milford, MA, United States) equipped with a Dikma ODS C_18_ column (150 × 4.6 mm, 5 μm) was used for separation at 30°C. The binary mobile phase consisted of A (an aqueous solution containing 0.1% acetic acid and 2 mM ammonium acetate) and B (acetonitrile) at a flow rate of 1 mL/min. The elution settings were as follows: 0 → 5 min: 10% B → 20% B; 5 min → 20 min: 20% B → 50% B; 20 min → 21 min: 50% B → 60% B; 21 min → 25 min: 60% B → 90% B; 25 min → 25.1 min 90%–10% B; 25.1 min → 30 min 10% B. Flow rate: 1 mL/min 260 nm was used as the detection wavelength. Calibration standard curves were generated using a reaction mixture containing tectorigenin, irigenin, or irisflorentin at a concentration range of 0.03–10 µM. The concentrations of the metabolites were calculated using a standard curve of the substrates.

### 2.7 Identification of the metabolites

A UPLC system coupled with an LTQ-Orbitrap mass spectrometer (Thermo Fisher Scientific, Waltham, MA, United States) was used. The mass spectrometer was equipped with an electrospray ionization source operating in negative ion mode for tectorigenin and irigenin, and in positive ion mode for irisflorentin. The following parameter settings were used: sheath gas flow rate, 45 arb; auxiliary gas flow rate, 15 arb; capillary temperature, 350°C; spray voltage, 3.8 kV; and heating component temperature, 300°C. Xcalibur software (Thermo Fisher Scientific) was employed to acquire and process the data.

### 2.8 Molecular docking

The crystal structures of human CYP1A2 (PDB: 2HI4), CYP2C19 (PDB: 4GQS), CYP2E1 (PDB: 3T3E), and CYP3A4 (PDB: 8SO1) were used as receptors and obtained from the Research Collaboratory for Structural Bioinformatics Protein Data Bank. Structures of tectorigenin, irigenin, and irisflorentin were constructed using Chem3D software, and the MM2 molecular force field was applied for energy minimization. Molecular docking was performed using AutoDock Vina software. A grid size of 60 × 60 × 60 Å with 0.375 Å spacing was centered on the co-crystallized ligand of each CYP protein. The Lamarckian genetic algorithm was employed for docking simulations, conducting a total of 100 runs, 2.5 × 10^6^ energy evaluations, and 27,000 iterations. All other parameters were set to their default values. The docking pose was selected based on the scoring function and protein–ligand interactions. PyMOL 2.0.6 was used for structural analysis.

### 2.9 Data analysis

GraphPad Prism software 5.0 was used to perform non-linear fitting between the metabolite generation rate (V) and substrate concentration [S]. The appropriateness of the enzyme kinetic model was determined using *R* ^2^ and the Eadie-Hofstee fit. The maximum metabolic rate (V_max_) and Michaelis constant (K_m_) were obtained for kinetic processes that conformed to the Michaelis–Menten model (Equation 1). For kinetic processes that conform to the sigmoidal kinetics model (Equation 2), V_max_, Hill coefficient (n), and S_50_, concentrations at 50% V_max_ were obtained. Intrinsic clearance (Cl_int_) was calculated according to Equation 3 (for the Michaelis–Menten model) and Equation 4 (for the sigmoidal kinetics model). All data are expressed as mean ± Standard Error (SE). One - way analysis of variance was used to compare the differences between different groups. A *p*-value below 0.05 was regarded as statistically significant.

$$V=\frac{V_{\max}S}{K_{m}+S}$$ (1)

$$V=\frac{V_{\max}+C^{n}}{S_{50}^{n}+C^{n}}$$ (2)

$${\text{Cl}}_{\mathrm{int}}=\frac{V_{\max}}{K_{m}}$$ (3)

$$Cl_{\mathrm{int}}=\frac{V_{\max}}{S_{50}}\times \frac{n-1}{n{\left(n-1\right)}^{1/n}}$$ (4)

## 3 Results

### 3.1 Metabolic stability and characterization of metabolites

**FIGURE 1:**
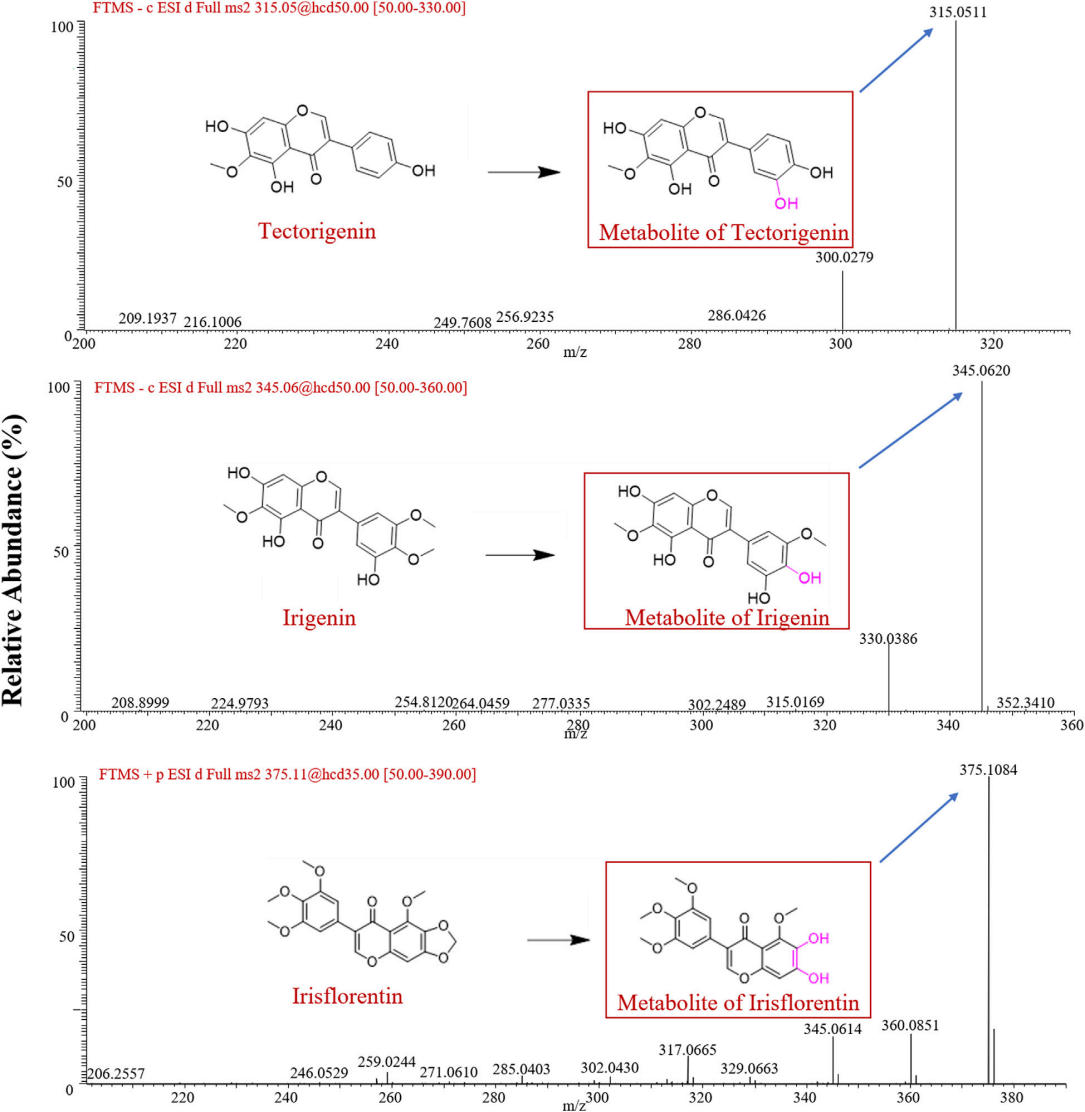
Mass spectra of the metabolites obtained in negative ion mode for tectorigenin and irigenin, and in positive ion mode for irisflorentin.

After incubation of tectorigenin with HLMs, a major metabolite with an exact molecular weight of 315.0511 Da in negative ion mode was identified as the hydroxylation metabolite of tectorigenin (Shi et al., 2015). The major metabolite of irigenin has an exact molecular weight of 345.0620 Da in the negative ion mode, which was speculated to be the demethylated metabolite of irigenin (Hu et al., 2021). For irisflorentin, the metabolite in the positive ion mode had an exact molecular weight of 375.1084 Da, and its fragment ions were 360.0851 Da and 345.0614 Da, respectively. Based on literature and mass spectrometry data, this compound was identified as 6,7-dihydroxy-5,3′,4′,5′-tetramethoxy isoflavone (Zhang Y. et al., 2021). The mass spectrometry data are shown in Figure 1. The red box in Figure 1 indicates the possible chemical structures of the metabolites.

### 3.2 Results of the chemical inhibition studies

**FIGURE 2:**
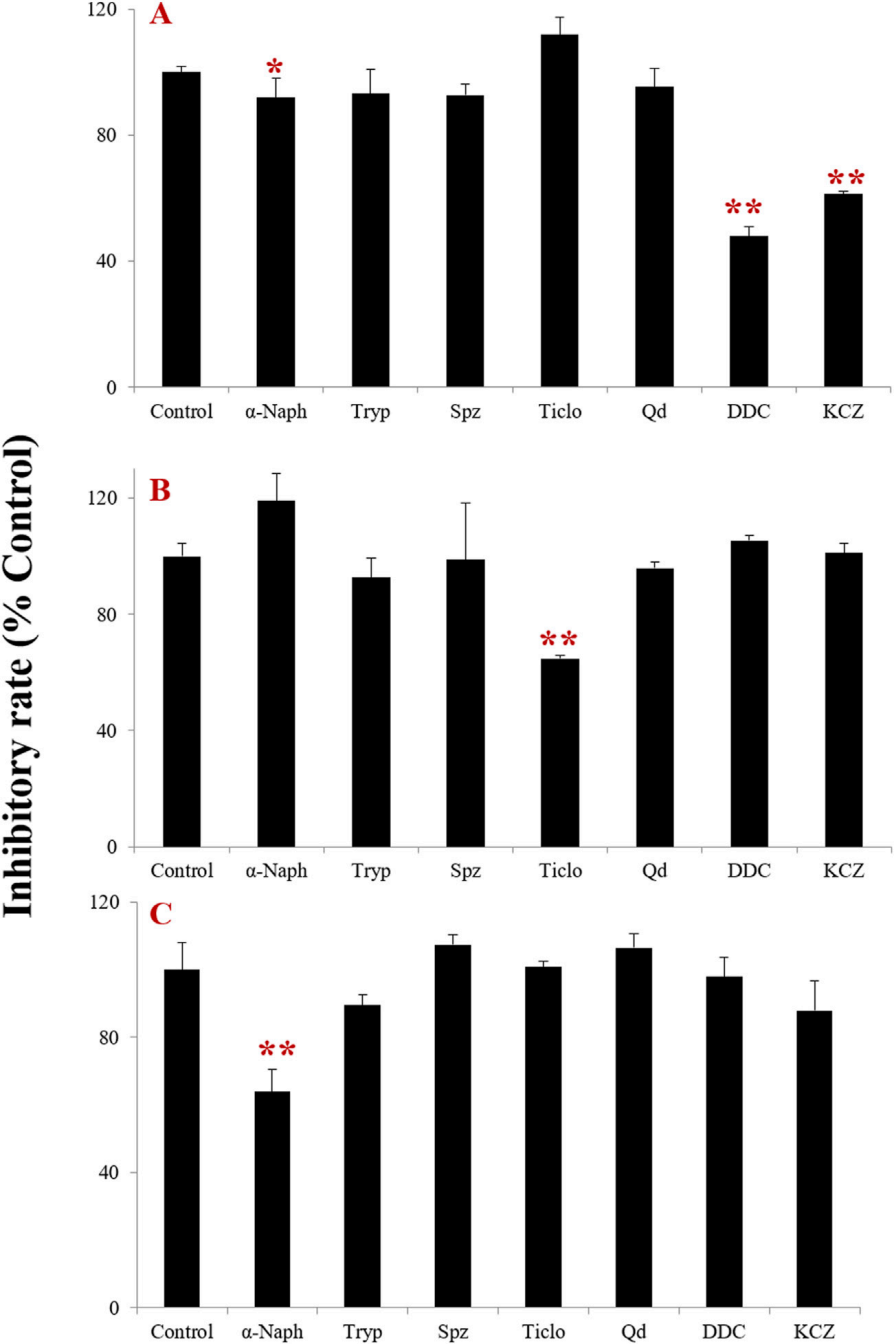
Effect of CYP chemical inhibitors on the formation rate of metabolites of tectorigenin **(A)**, irigenin **(B)**, and irisflorentin **(C)** with HLMs (mean ± SE, n = 3). *: *p* < 0.05, **: *p* < 0.01. HLMs, human liver microsomes.

The effects of selective inhibitors of the major CYP isoenzymes involved in tectorigenin, irigenin, and irisflorentin metabolism in HLMs are shown in Figure 2. With an inhibition rate exceeding 15.0% as the threshold, it was found that the specific inhibitors DDC (the inhibitor of CYP2E1) and KCZ (the inhibitor of CYP3A4) could significantly inhibit the formation of tectorigenin metabolite with the inhibition rates of 52.1% and 38.6%, respectively. And Spz (a CYP2C9 inhibitor) slightly inhibited the metabolite formation of tectorigenin with 7.6%. Ticlo for CYP2B6 and 2C19 could significantly inhibit the formation of irigenin metabolites at an inhibition rate of 35.2%. And α-Naph (a CYP1A2 inhibitor) could significantly inhibit the formation of irigenin metabolites (the inhibition rate of 36.1%). These results suggest that these three compounds are metabolized by different CYP enzymes in HLMs.

### 3.3 Metabolism by recombinant CYP enzymes

**FIGURE 3:**
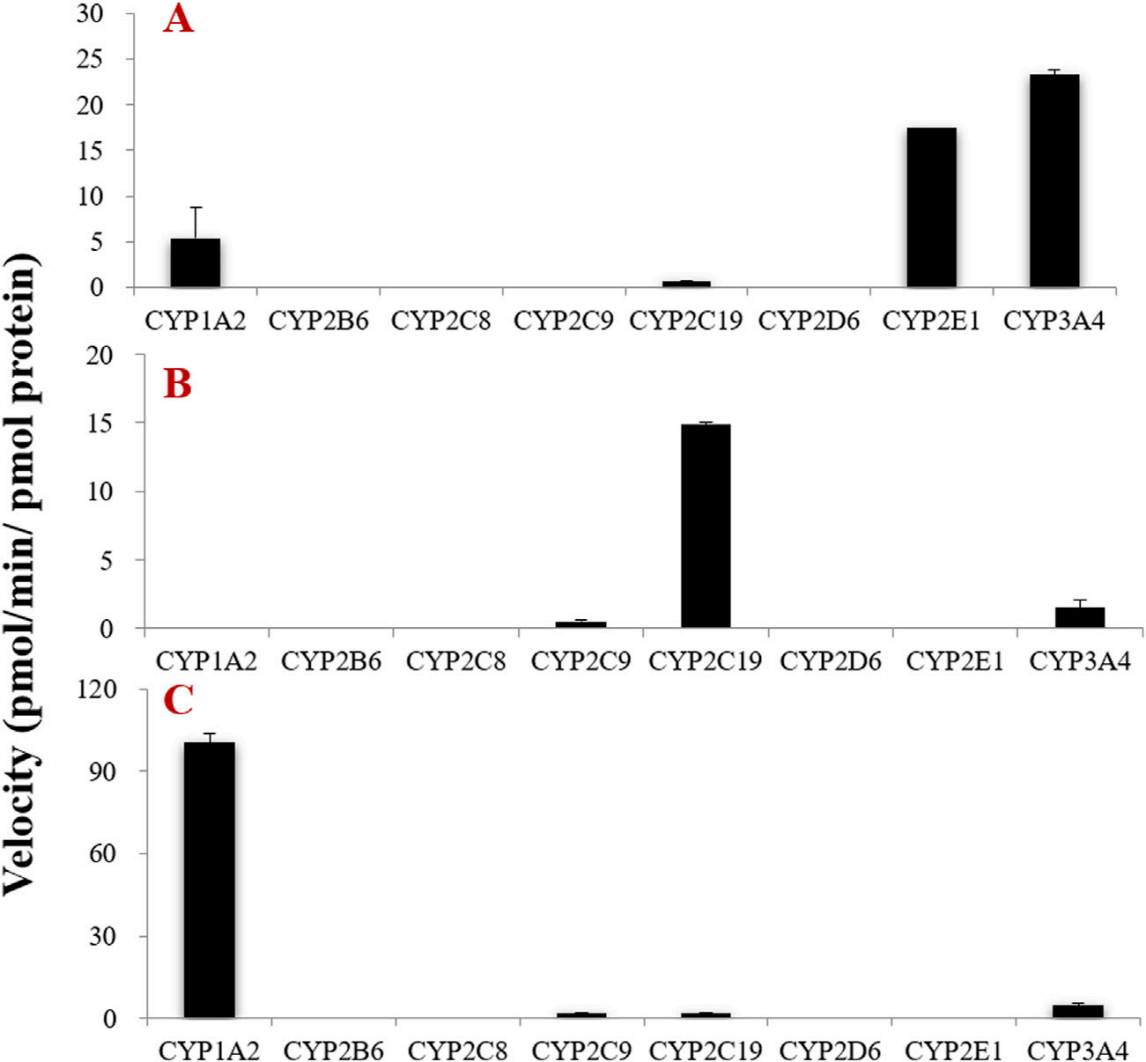
Formation rates of tectorigenin **(A)** irigenin **(B)** and irisflorentin **(C)** metabolites by recombinant CYP enzymes (mean ± SE, n = 3).

To further determine the CYP subtypes that mediate the phase I metabolism of tectorigenin, irigenin, and irisflorentin, we used eight commercially available recombinant CYP enzymes. The results showed that among the eight CYP subtypes measured, CYP1A2, CYP2C19, CYP2E1, and CYP3A4 catalyzed the metabolic reaction of tectorigenin (Figure 3A). Among these, CYP2E1 and CYP3A4 showed the highest metabolic rates. CYP3A4, CYP2C9, and CYP2C19 are involved in the formation of irigenin metabolites (Figure 3B). Among the three recombinant CYP enzymes, CYP1A2, CYP2C19, and CYP3A4, CYP1A2 catalyzed the generation of the largest number of metabolites (Figure 3C).

### 3.4 Enzyme kinetic properties in HLMs

**FIGURE 4:**
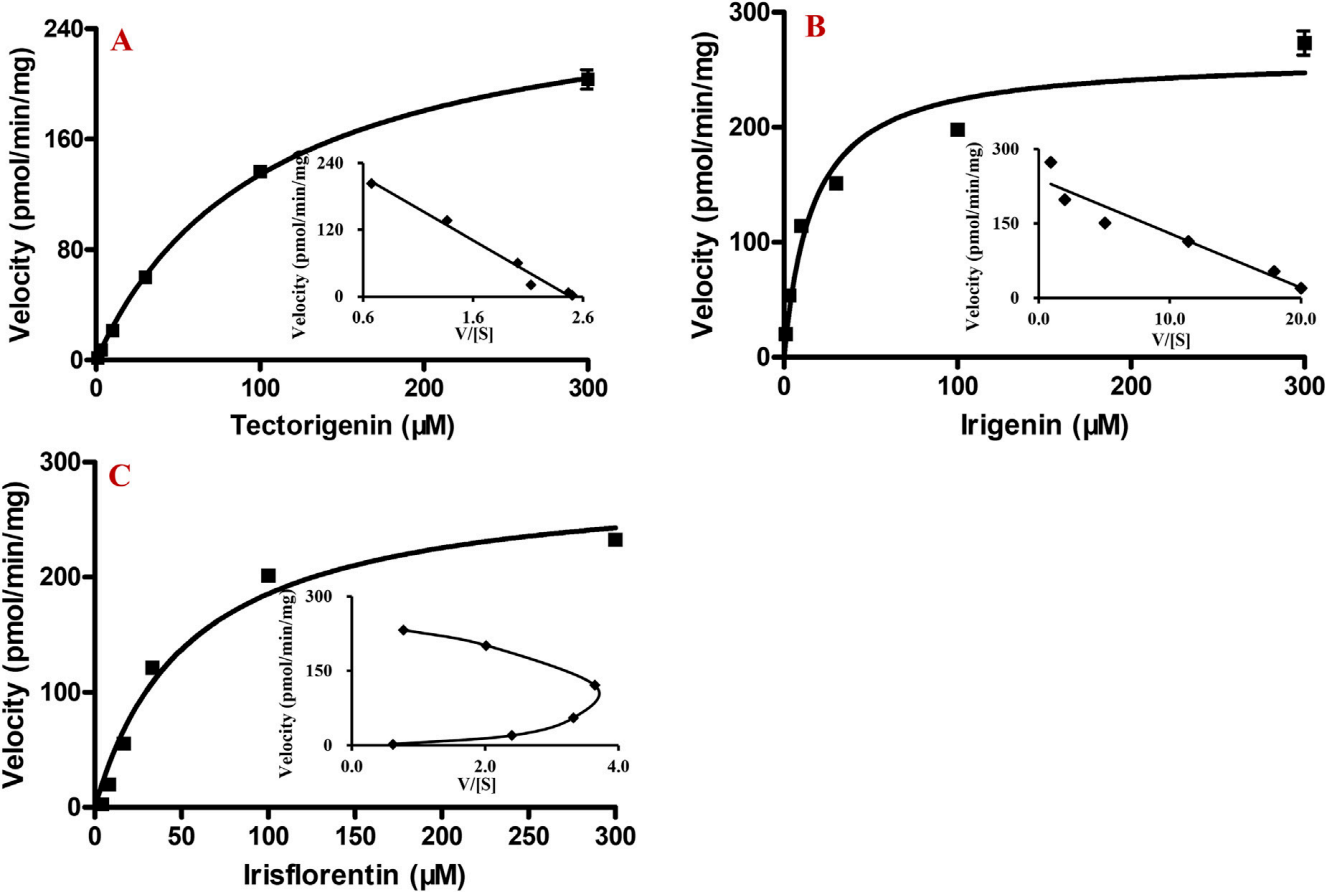
Kinetics of tectorigenin, irigenin and irisflorentin with HLMs (mean ± SE, n = 3). **(A)** tectorigenin. **(B)** irigenin. **(C)** irisflorentin. HLMs, human liver microsomes.

After incubation of HLMs with different concentrations of tectorigenin, irigenin, or irisflorentin, the production of metabolites increased with increasing substrate concentration. From the Eadie-Hofstee plot (Figures 4A, B, inset), it was found that the enzymatic kinetic processes of tectorigenin and irigenin conformed to the Michaelis–Menten equation. K_m_, V_max_, and Cl_int_ for metabolite formation of tectorigenin were 105.5 ± 7.3 μM, 275.9 ± 7.6 pmol/min/mg protein, and 2.6 μL/min/mg protein, respectively, whereas those for metabolite formation of irigenin were 17.5 ± 3.2 μM, 263.1 ± 13.6 nmol/min/mg, and 15.0 μL/min/mg protein, respectively.

The Eadie-Hofstee plot of irisflorentin yielded a convex curve with a unique “hooked” characteristic, which conformed to the sigmoidal kinetics characteristic (Figure 4C, inset). The S_50_ and V_max_ were 32.9 ± 1.0 μM and 235.4 ± 3.2 pmol/min/mg protein, respectively. The Hill coefficient (n) and Clint were 1.8 ± 0.1 and 3.6 μL/min/mg protein, respectively.

### 3.5 Enzyme kinetic properties of recombinant CYP enzymes

**FIGURE 5:**
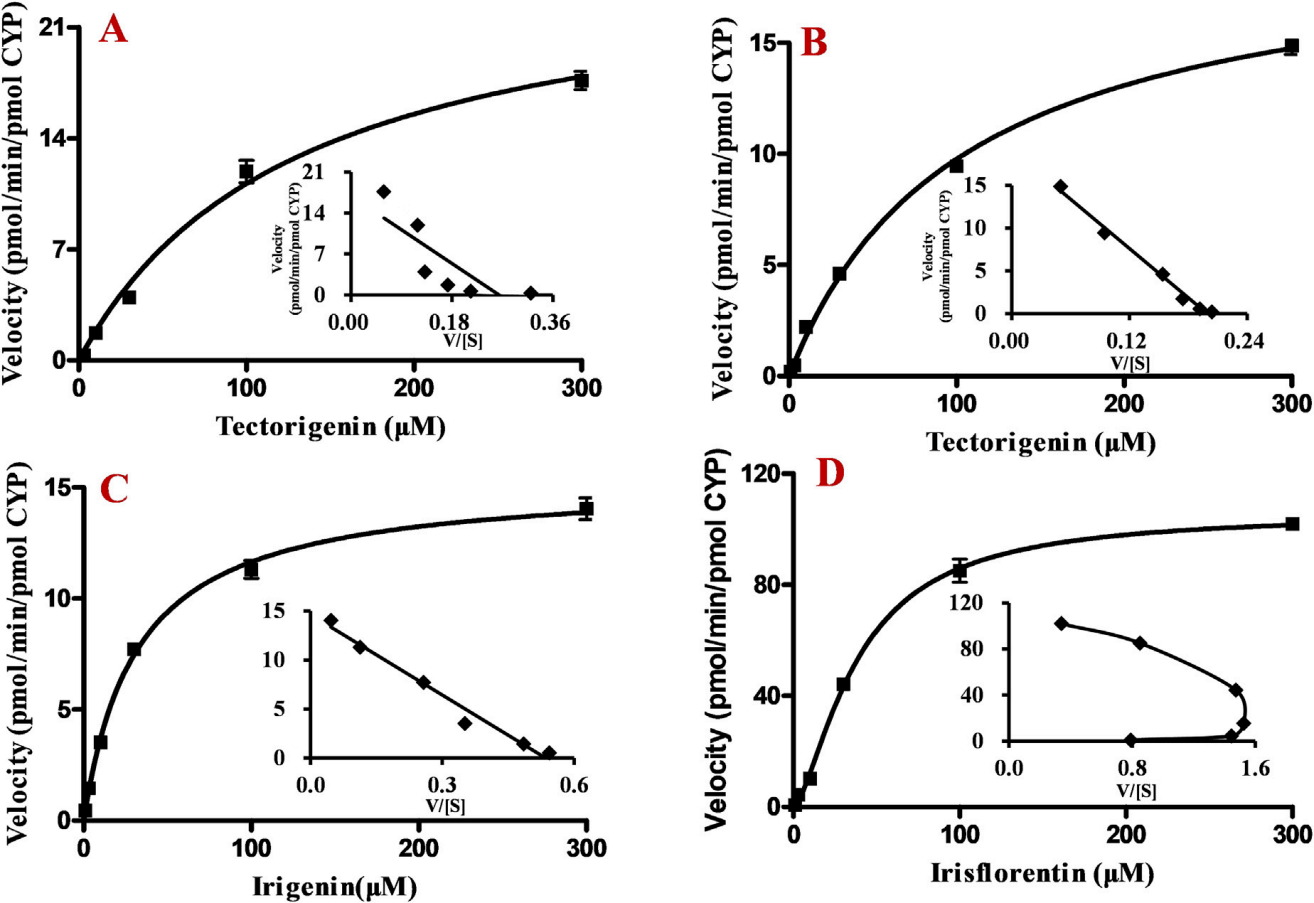
Enzyme kinetic properties of recombinant CYP enzymes. **(A)** Tectorigenin with CYP2E1. **(B)** tectorigenin with CYP3A4. **(C)** irigenin with CYP2C19. **(D)** irisflorentin with CYP1A2.

The kinetics of the main recombinant CYP enzymes involved in tectorigenin, irigenin, and irisflorentin metabolism were further investigated (Figure 5). From the Eadie-Hofstee plot (Figure 5, inset), it could be found that the kinetic processes of tectorigenin and irigenin by the recombinant enzymes both conform to the Michaelis-Menten equation (Figures 5A–C), while the enzymatic kinetic process of irisflorentin conformed to the sigmoidal kinetics characteristic with a Hill coefficient of 1.5 ± 0.1 (Figure 5D). The kinetic parameters of these enzymes are showed in Table 1.

**TABLE 1:** Enzyme kinetic parameters of tectorigenin, irigenin, and irisflorentin with recombinant CYP enzymes (mean ± SE, n = 3).

Substrate	Enzyme	K_m_/S_50_	V_max_	Clint
		(μM)	(pmol/min/pmol CYP)	(μL/min/pmol CYP)
Tectorigenin	CYP2E1	130.4 ± 18.8	25.6 ± 1.6	0.2
	CYP3A4	102.9 ± 7.5	19.8 ± 0.6	0.2
Irigenin	CYP2C19	31.7 ± 2.4	15.3 ± 0.3	0.5
Irisflorentin	CYP1A2	38.5 ± 2.5	106.0 ± 2.8	1.5

### 3.6 Molecular docking simulation

**FIGURE 6:**
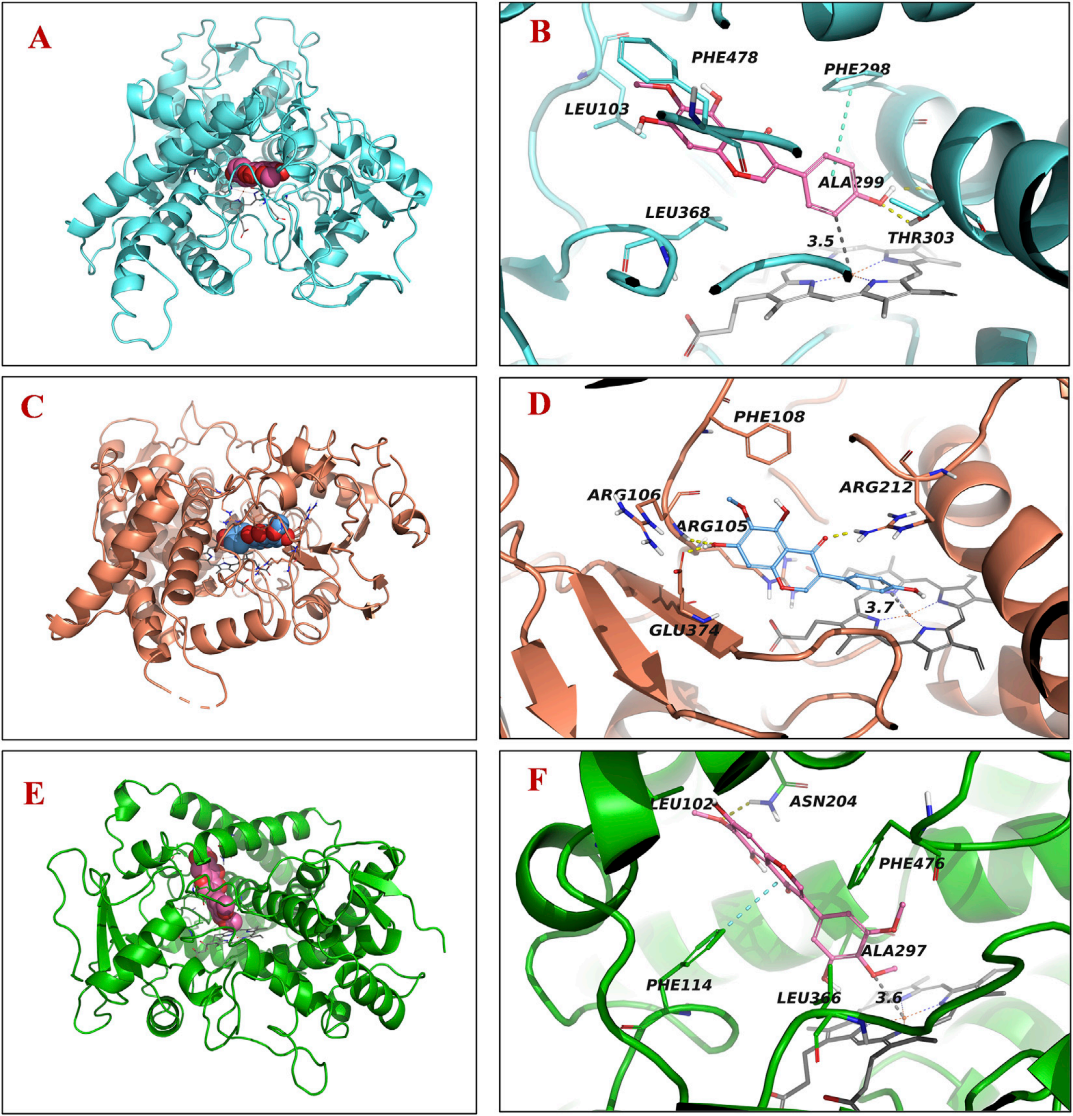
Molecular docking analysis demonstrating binding positions of tectorigenin and irigenin to human CYP enzymes. Three-dimensional illustrations for tectorigenin with CYP2E1 **(A)** and CYP3A4 **(C)** and irigenin with CYP2C19 **(E)**. Two-dimensional diagrams for tectorigenin with CYP2E1 **(B)** and CYP3A4 **(D)** and irigenin with CYP 2C19 **(F)**.

The molecular docking results showed that tectogenin binds to the orthosteric site of CYP2E1 (PDB: 3T3Z), with a high binding affinity of −8.1 kcal/mol, forming hydrogen bonds and π-π stacking with Thr303 and Phe298, respectively. The benzene ring interacted with Fe^2+^ (d = 3.5 Å) of heme. Tectogenin displayed a high binding affinity of −7.9 kcal/mol at the active site of CYP3A4 (PDB: 8SO1). It formed hydrogen bonds with Arg105, Arg106, and Arg212, with the benzene ring interacting with Fe^2+^ (d = 3.7 Å) of heme. Therefore, we speculated that the oxidation of benzene rings was the main metabolic pathway of tectogenin, catalyzed by CYP2E1 and CYP3A4. Irigenin could bind to the orthosteric site of CYP2C19, forming hydrogen bonds with Asn204 and π-π stacking with Phe114, with a high binding affinity of −7.5 kcal/mol. The methoxy group interacted with Fe^2+^ (d = 3.6 Å) of heme, suggesting that CYP2C19 catalyzes the metabolic reaction of irigenin, leading to the formation of demethylated products. The binding positions of tectorigenin and irigenin are shown in Figure 6.

**FIGURE 7:**
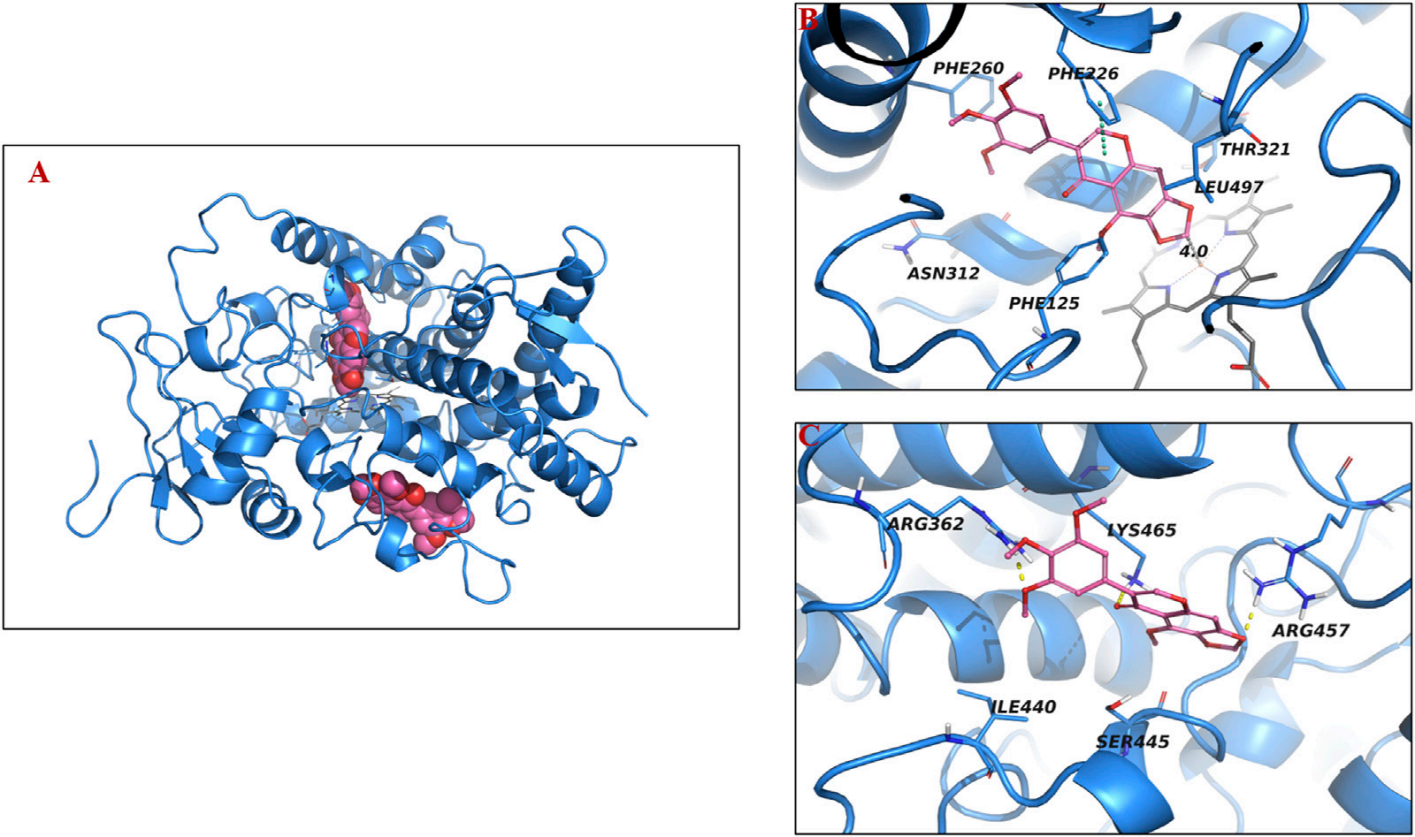
Molecular docking analysis demonstrating binding positions of irisflorentin in human CYP1A2. Three-dimensional illustration **(A)**, two-dimensional diagrams for substrate pocket **(B)** and allosteric binding site **(C)**.

As shown in Figure 7, irisflorentin could potentially bind to both binding pockets of the CYP1A2 protein (PDB: 2HI4), specifically the substrate pocket and the allosteric binding site, with binding affinities of −8.2 kcal/mol and −6.4 kcal/mol, respectively. In the substrate-binding pocket, irisflorentin formed π-π stacking with Phe226 and hydrophobic interactions with Phe260, Asn312, Phe125, Leu497, and Thr321. The carbon in the five-membered ring was aligned with the Fe^2+^ (d = 4.0 Å) of heme, suggesting that CYP1A2 oxidizes the carbon atom, causing the five-membered ring to open and generate 6,7-dihydroxy-5,3,4,5-tetramethoxy isoflavone.

## 4 Discussion

Understanding the pharmacokinetic characteristics *in vivo* of these components is of great significance for their improved clinical use. In our previous *in vitro* studies, tectorigenin and irigenin were widely metabolized by UDP-glucuronosyltransferase enzymes (Li et al., 2022). Some researchers have also found through *in vivo* studies that these flavonoid components can produce phase I metabolites that are deoxygenated, demethoxylated, and demethylated. However, the phase I pharmacokinetic characteristics of these components have not yet been fully elucidated.

The liver serves as the principal site of metabolism and contains various CYP metabolic enzymes. Conducting metabolic research using an *in vitro* liver microsome incubation system not only eliminates the interference of many factors *in vivo* but also effectively solves key problems in metabolism, such as metabolite structure identification and metabolic pathway inference, providing important clues and a basis for *in vivo* metabolism research. Therefore, in this study, we used HLMs to evaluate the stability of these three compounds. The results showed that after the co-incubation of the three components with liver microsomes, a major metabolite was generated for each component. For tectorigenin, we detected a metabolite ion with m/z [M-H]^+^ of 315 (an increase of 16 Da compared to tectorigenin), a metabolite formed by the hydroxylation of tectorigenin, which is similar to that reported in an *in vivo* study. Shi et al. (2015) found that after intragastric administration of tectorigenin (65 and 130 mg/kg) in rats, multiple tectorigenin metabolites were observed *in vivo*. Among these metabolites, in addition to the directly conjugated metabolites formed by tectorigenin and glucuronide or sulfate, it was also found that the ions with the parent ion [M + H]^+^ of m/z 573.0547, 493.0681, 476.9785, and 397.0236 could generate fragment ion at m/z 317.06 (an increment of 16 Da compared to tectorigenin) by hydroxylation of tectorigenin. Therefore, metabolites generated *in vitro* are important *in vivo* phase I metabolites. For irigenin, we have confirmed by mass spectrometry that the molecular weight of the newly generated metabolite has lost 14 Da compared to the prototype in HLMs. Although no previous studies have directly reported irigenin metabolites, Hu et al. (2021) studied the metabolites of iridin (the glycoside of irigenin) in the plasma and urine. Glycosides are first hydrolyzed by the intestinal flora to generate aglycones in the intestine, which then enter the body and are metabolized further. Among the generated metabolites, there was a demethylated metabolite of irigenin at m/z [M + H]^+^ of 347.0749 (a loss of 14 Da compared to that of irigenin). The finding of this metabolite accorded with the results of a previous study, suggesting that demethylation is an important phase I metabolic reaction of irigenin. A previous study reported that when irisflorentin is co-incubated with rat liver microsomes, multiple metabolites can be detected, among which 6,7-dihydroxy-5,3′,4′,5′-tetramethoxyisoflavone at m/z 375 [M + H]^+^ is the main metabolite (Zhang Y. J. et al., 2021). Our main metabolite exhibited a parent ion at m/z 375 [M + H]^+^, which had a molecular weight 12 Da less than that of irisflorentin, indicating the breaking of the methylene acetal group of irisflorentin, which was consistent with the product generated in human and rat liver microsomes (Jia et al., 2016). Studies had confirmed that the metabolite named 6,7-dihydroxy-5.3′,4′,5′-tetramethoxy isoflavone originated irisflorentin had much higher pharmacological activity than its parent compound (Jia et al., 2016; Wang et al., 2024). Therefore, the metabolism study had the great significance for better understanding the biological effects of iridoid flavonoids *in vivo*.

To identify the metabolic pathways, we used two methods: chemical inhibitors in HLMs and recombinant CYP enzymes to explore the key enzymes that catalyze the metabolism of these compounds. α-Naph, the CYP1A2 inhibitor, had a slight inhibition on the demethylation of tectorigenin and a strong inhibition on the breaking of methylene acetal group of irisflorentin. Ticlo, a specific inhibitor of CYP2B6 and 2C19, significantly inhibited the formation of irigenin metabolites but had no effect or a weak inhibitory effect on the generation of tectorigenin and irisflorentin metabolites. The CYP2E1 DDC inhibitor only affected the generation of tectorigenin metabolites. The selective inhibitor of CYP3A4, KCZ, affected the metabolism of tectorigenin but had relatively little impact on the other two components. The effects of these inhibitors on metabolite production were verified using recombinant enzymes. The production of metabolites mediated by CYP2E1 and CYP3A4 was 3–38 times higher than that of the other subtypes. CYP2C19 catalyzes irigenin metabolism, which is 10–29 times higher than that of CYP2C9 and CYP3A4. The ability of CYP1A2 to participate in the metabolism and transformation of irisflorentin was 21–55 times higher than that of CYP2C9, CYP2C19, and CYP3A4. These results suggest that CYP3A4 (tectorigenin), CYP2E1 (tectorigenin), CYP2C19 (irigenin), and CYP1A2 (irisflorentin) are the most critical metabolic enzymes catalyzing these three components. As a supplement to *in vitro* experimental research, with the help of molecular docking, we further observed the binding capacity and specific interactions between isoflavone aglycone and CYP2E1, CYP3A4, and CYP1A2 isozymes and inferred the most likely chemical structure of metabolites from the optimal spatial binding posture.

In enzyme kinetic studies, the Eadie-Hofstee plot showed that the velocity and velocity/substrate concentrations of tectorigenin and irigenin formed a linear relationship. Therefore, regardless of whether they are metabolized by HLMs or recombinant enzymes, their enzyme kinetics conform to the Michaelis-Menten process. The K_m_ and V_max_ values of CYP2E1 and CYP3A4 for tectorigenin metabolism did not differ significantly. CYP3A4 is the predominant human -metabolizing enzyme, making up about 20%–30% of the total CYP in the liver, with CYP2E1 (15%–25%) coming next (Liu et al., 2021); therefore, it is speculated that *in vivo* CYP3A4 and CYP2E1 both contributed significantly to the generation of the metabolite of tectorigenin. From the reported plasma and liver data, the maximum concentration of these components ranged from tens to hundreds of ng/mL (Xiong et al., 2024) after administration of Shegan (10.5 g/kg) were far below the values of K_m_, indicating that the formation rate of metabolites is directly proportional to the substrate concentration *in vivo* (Zhang et al., 2007). Compared with the clearance of tectorigenin and irigenin during phase II metabolism conducted previously (Li et al., 2022), the clearance rates of phase I metabolism in HLMs were significantly lower. This observation aligns with the detection of a wide range of conjugated metabolites *in vivo*, likely because the exposed phenolic hydroxyl groups can readily conjugate with glucuronic acid or sulfate to form phase II metabolites (Shangari et al., 2005).

In general, metabolic enzymes may possess multiple substrate binding sites within their molecules, leading to diverse interactions such as positive cooperativity, negative cooperativity, and substrate inhibition (Liu and Liu, 2022). The self-activation characteristic of positive cooperativity is reflected in the kinetic curve, characterized by a slower rate of metabolite generation at low substrate concentrations and a rapid, exponential increase as substrate concentration rises (Hidayat et al., 2024). The convex, “hook-shaped” contour of irisflorentin suggests that it exhibits sigmoidal kinetics and positive cooperativity (Marques et al., 2014). Therefore, we employed the Hill equation—the most commonly used equation to describe sigmoidal kinetics—to calculate the enzymatic kinetic parameters of irisflorentin. The parameter “n” in the equation, known as the Hill coefficient, reflects the degree of cooperativity and the degree of sigmoidal behavior observed (i.e., the larger the value of *n*, the greater the cooperativity and sigmoidicity) (Zhang et al., 2007). Our results showed that the Hill coefficients in HLMs and recombinant CYP1A2 were 1.8 and 1.5, respectively. A similar phenomenon was observed during the co-incubation of irisflorentin with rat liver microsomes, with a Hill coefficient of 1.48 (Zhang Y. J. et al., 2021). The similarity of irisflorentin in human and rat liver microsomes suggests the possibility of extrapolating the pharmacokinetic characteristics of this compound across species. Notably, the nonlinear regression analysis curve of CYP1A2-catalyzed metabolic reactions did not conform to the classical Michaelis–Menten model, a pattern also observed in the data analysis of the other compound (Hidayat et al., 2024).

Because of the low production of phase I metabolites, we attempted to increase the concentration of the substrate and the reaction time (240 min) to collect the metabolites; however, we did not have sufficient pure components for quantitative analysis. Based on the similarity in the parent nuclear structure between the prototype components and metabolites, it was presumed that their ultraviolet absorption characteristics were relatively similar. Therefore, the standard curve of the prototype was used to semi-quantitatively evaluate the amount of metabolites generated according to methods described in the literature (Li et al., 2022).

## 5 Conclusion

To the best of our knowledge, this is the first study to reveal the *in vitro* metabolism of tectorigenin, irigenin, and irisflorentin in human. CYP2E1, CYP3A4, CYP2C19, and CYP1A2 are the most important enzymes responsible for the metabolism of these compounds, and their kinetic characteristics are either typical or atypical. These findings will enhance our understanding of the pharmacology of these compounds and promote the development and clinical application of this herb.

## Data Availability

The raw data supporting the conclusions of this article will be made available by the authors, without undue reservation.

## Abbreviations

α-Naph, α-naphthoflavone; Cl_int_, Intrinsic Clearance; CYP, Cytochrome P450; Qd, Quinidine; DDC, Diethyldithiocarbamate; KCZ, Ketoconazole; K_m_, Michaelis constant; HLMs, Human liver microsomes; NADPH, Nicotinamide adenine dinucleotide phosphate tetrasodium salt; [S], Substrate concentration; S_50_, Concentration at 50% of V_max_; SE, Standard Error; Spz, Sulfaphenazole; Ticlo, Ticlopidine hydrochloride; Tryp, Tryptamine; V, Metabolite generation rate; V_max_, Maximum metabolic generation rate.
